# A Queue-Based Monte Carlo Analysis to Support Decision Making for Implementation of an Emergency Department Fast Track

**DOI:** 10.1155/2017/6536523

**Published:** 2017-03-28

**Authors:** Kristin Fitzgerald, Lori Pelletier, Martin A. Reznek

**Affiliations:** ^1^Operations Research and Financial Engineering, Princeton University, Princeton, NJ 08540, USA; ^2^Center for Innovation and Transformational Change, UMass Memorial Health Care, Worcester, MA 01655, USA; ^3^Operational Excellence, UMass Memorial Health Care, Worcester, MA 01655, USA; ^4^Department of Emergency Medicine, University of Massachusetts Medical School and UMass Memorial Health Care, Worcester, MA 01655, USA

## Abstract

Emergency departments (EDs) are seeking ways to utilize existing resources more efficiently as they face rising numbers of patient visits. This study explored the impact on patient wait times and nursing resource demand from the addition of a fast track, or separate unit for low-acuity patients, in the ED using a queue-based Monte Carlo simulation in MATLAB. The model integrated principles of queueing theory and expanded the discrete event simulation to account for time-based arrival rates. Additionally, the ED occupancy and nursing resource demand were modeled and analyzed using the Emergency Severity Index (ESI) levels of patients, rather than the number of beds in the department. Simulation results indicated that the addition of a separate fast track with an additional nurse reduced overall median wait times by 35.8 ± 2.2 percent and reduced average nursing resource demand in the main ED during hours of operation. This novel modeling approach may be easily disseminated and informs hospital decision-makers of the impact of implementing a fast track or similar system on both patient wait times and acuity-based nursing resource demand.

## 1. Introduction

As health care reform is implemented across the United States, the total number of emergency department (ED) visits is rising [1, 2]. While evidence suggests that the Affordable Care Act may increase overall reimbursement for low-income cases, such as those covered by Medicaid or the uninsured [3], the growth in visits has consequences beyond the financial. Hospitals are challenged to serve an increasing number of patients under limited resources and rising costs, making it a priority to use resources effectively when caring for patients. With more patients arriving at the ED, hospitals observe increased wait times and increased patient length of stay [4]. However, other negative effects include delay in care, decreased patient satisfaction, and increased mortality [5, 6]. Other observable effects include patient beds in the hallways and high nurse-to-patient ratios. Overall, these effects lower the quality of care a hospital has to offer. When patients perceive a lower quality of service or longer waiting times, they are more likely to leave the ED without being seen (LWBS) and go to other hospitals, resulting in negative perceptions of the hospital and health risks for the patient [7–9].

The adoption of separate units within the ED for low-acuity patients, often referred to as “fast tracks,” has been supported by experts and national organizations within emergency medicine [10–12]. Addition of a fast track has also been reported to increase patient satisfaction [13]. For hospitals that use the ESI scoring for triage, most patients triaged with an ESI level 4 or 5 are evaluated and treated in the fast track, while other patients with more severe cases are seen in the main ED [14, 15]. While these fast tracks have been implemented in many EDs nationwide, scientific information remains limited with regard to specific criteria and methods to effectively evaluate the need for fast track implementation within a specific ED [10]. Issues such as total ED patient volume, proportion of low-acuity patients, optimal time of day for implementation, and cost-benefit analysis have been cited as specific areas lacking in the current body of research [10].

Given the limitations of available published data, and given the flexibility of computer simulation, we felt that modeling would provide an important mechanism to support decision making regarding fast track implementation in one of our hospitals. We developed a simulation model that expands upon the fundamental queueing model to account for various real-life factors such as priority queueing and dynamic arrivals, in order to create an accurate model of ED flow with or without the addition of a fast track. Additionally, our simulation models ED capacity by incorporating patients' Emergency Severity Index (ESI) levels [16] and the number of nurses on each shift to determine current occupancy and available capacity. This essential architecture allows the simulation to evaluate the nursing resource demand within the department from an overall patient severity perspective, rather than the number of beds occupied, which is a typical variable in most ED capacity models [10, 17, 18]. Of note, unlike nursing in our ED, licensed independent providers—physicians and advanced practice providers (APPs)—were less resource constrained at the time of modeling. ED leadership was confident that there was capacity in this group to accommodate more patients and that nursing was the constrained personnel resource. Nursing was therefore the personnel factor evaluated in this particular model; other personnel constraints such as providers may be evaluated at a later time or another institution by adjusting the resources in the simulation.

Queueing theory is the mathematical modeling of queues or waiting lines [19]. It has frequently been utilized in hospital analysis and simulation, as its simple structure easily evaluates arrivals, waiting times, and service times [17, 20, 21]. Queueing theory may be combined with Monte Carlo simulation or discrete event simulation to produce numerical results for complex models [19]. However, simple queueing models do not account for dynamic arrival rates, different service times, and other characteristics of the ED.

A variety of other simulation techniques have also been used to model and evaluate ED fast tracks. A 2015 study used an agent-based simulation, which models the actions/interactions of independent agents, to evaluate fast track strategies in order to reduce wait times in a hospital ED [22]. Discrete event simulation, which models the system as a series of distinct events over time, was used in a 1995 study to model the impact of a fast track on patient wait times [18]. A similar discrete event simulation was used in a 2008 study to evaluate buffer concepts such as a fast track [23]. While these studies have successfully modeled a fast track in the ED, they involved building highly complex models that lack the simplicity of a queueing system. They also do not report the impact on health care provider resource demand, particularly as it relates to patient acuity.

For our simulation case site, the addition of a separate fast track reduced wait times for low-acuity patients with little or no impact on the wait times of other patients and without increasing nursing resource demand. Beyond this study, we believe that the flexibility of the model's discrete event architecture and its basis in queueing theory enables it to be applied to other EDs with different staffing numbers, arrival rates, and/or service times. Additionally, the model enabled us to optimize the fast track hours of operation, staffing requirements, and nursing resource demand to have the largest impact on patient wait times without negatively affecting the quality of service.

## 2. Methods

### 2.1. Setting and Data Sources

The case site of this study was a two-campus, private, nonprofit medical center in central Massachusetts. Between the two campuses, there are over 750 licensed beds and almost 4000 active medical staff and nurses. The two EDs together see over 130,000 patients per year. The smaller one of the two campuses, campus A, accounts for approximately 40 percent of the total ED visits. The ED at campus B opened a unit dedicated to low-acuity patients, and campus A was interested in the implications of opening a similar fast track unit, “East Pod,” multiple days a week. The simulation model was built to model the ED at campus A.

We obtained deidentified data from the hospital ED's electronic health record (EHR) and management tool (Picis ED PulseCheck, Wakefield, MA), including major timestamps for each patient during their visit in addition to the patient's recorded ESI level. Relevant timestamps included time of arrival, arrival time in the room, and time of departure. During data abstraction and validation, we excluded duplicate records, patients who were dead on arrival (DOA), and records that did not contain arrival time, departure time, or ESI level. These exclusions accounted for less than 1% of data received. This study was approved by the Princeton Institutional Review Board (Princeton, NJ) (Princeton University IRB no. 7347).

### 2.2. Model Design

Prior to model building, we performed descriptive statistics on the patient records to determine average arrival rates by ESI, hour of day, and day of week using statistical functions in MATLAB. The distributions of current wait and service times were also computed for each ESI. Additionally, we evaluated the relationship between LWBS rate and time spent in the waiting room using regression models.

The model integrated elements of discrete event simulation with queueing theory to more accurately represent the variations in a hospital ED. It was built in MATLAB due to the programming language's ease of vector and matrix operations, simple data importation/exportation tools, and native support of advanced mathematical functions. However, the authors acknowledge that other programming languages may be utilized to build similar models due to the discrete nature of the queueing model. Please see Appendix A for more details regarding this particular model structure in MATLAB.

**Figure 1:**
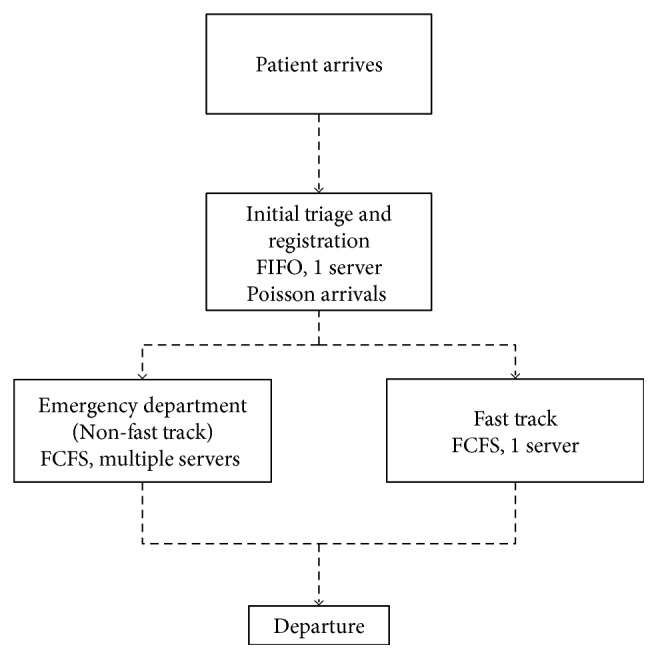
Emergency department queueing system.

A multiphase queueing system best described the patient flow process. Each patient first entered a triage/registration queue (first-in, first-out) and was then put into either the regular ED queue or the fast track queue (first-come, first-served) as shown in Figure 1. Arrivals followed a Poisson process, which is described in detail in Appendix B.

Patients in the main ED queue were admitted by priority, lowest ESI first. The model assumed that priority is always maintained, regardless of the total wait times of patients in the queue.

To model nursing resource demand, the simulation assumed that there are five times as many service providers available as there are nurses. The ESI score of a patient determined the number of providers he/she needs (see Table 1). These ratios were determined through staff feedback.

Upon each patient's arrival, the model determined the number of required providers and the earliest time that the number of providers is available. Providers were made available after the previous patient departed the ED plus a short delay for cleaning purposes. If the time of availability was after the patient's arrival, they were added to a waiting queue, which was admitted by priority.

**Figure 2:**
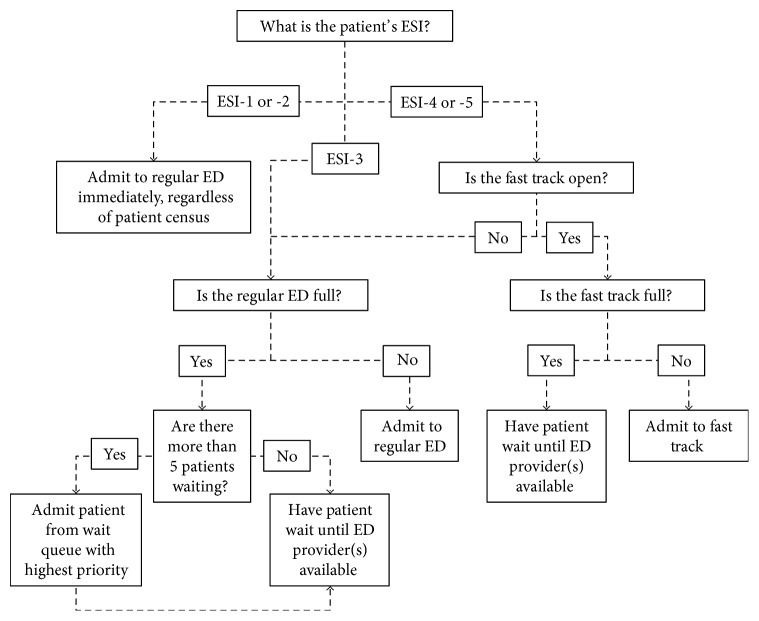
Model decision tree for ED/fast track admission.

If a patient had an ESI level of 1 or 2, or if there were more than 5 patients in the waiting queue, the number of providers available was increased to admit the patient with highest priority, as indicated by staff feedback and trends in historical data. When the patient left, the number of providers in the model returned to its original state. See Figure 2 for the model's decision process for admission.

The model used a left without being seen (LWBS) regression fit from campus A's historical data for wait times to model a patient's probability of LWBS.

A variety of simulation scenarios were evaluated, exploring variable nurse scheduling, fast track hours of operation, and fast track days of operation. The “current state” simulation used the current nurse schedule without a fast track in operation.

The primary outcome metrics for this study were patient wait time and nursing resource demand.

## 3. Results

### 3.1. Current State Analysis

Campus A saw 39148 ED visits from June 1, 2014, to May 31, 2015. Approximately 57 percent of patients were female, and 1.70 percent of these patients were LWBS. The median recorded wait time was less than 5 minutes for non-LWBS patients, but nearly 75 minutes for LWBS patients. The median overall length of stay was 3.78 hours. Approximately 30 percent of all patients have ESI levels of 4 or 5 and are fast track eligible. The percentage of LWBS patients was the highest among ESI-4 and ESI-5 patients. See Table 2 for details.

**Figure 3:**
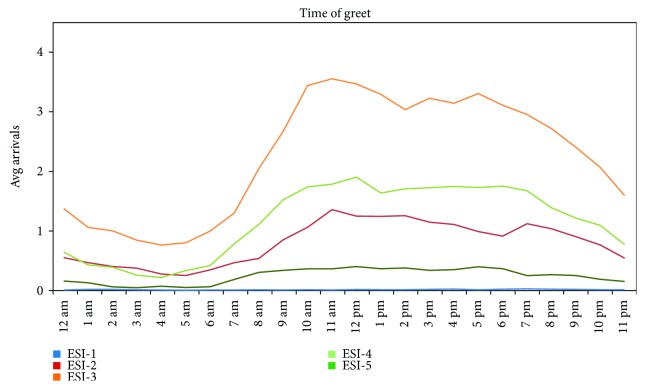
Average hourly arrivals by ESI for all days (campus A).

Campus A hourly arrivals followed a similar trend across all ESI levels (Figure 3). Peak hours of operation ranged from 9:00 am to 5:00 pm, and weekdays saw more patients on average than weekends.

### 3.2. Monte Carlo Analysis

The model generated 300 sets of simulation data for each simulation scenario (see Table 3 for an abbreviated list of scenarios). Additional, nonsignificant scenario results are not discussed here for the sake of brevity. For each set, consisting of 365 days worth of arrivals, the model recorded the median wait time, maximum wait time, and LWBS rate.

A Student *t*-test (level of significance = 0.05) verified that each simulation scenario exhibited statistically significant median wait times from the current state. There was no significant difference between observed maximum wait times. While there was a significant difference between LWBS rates of the current states and the other simulation scenarios, the current LWBS rate of the hospital was low enough that any impact was negligible and highly variable.

The model's wait time results varied from the historical data due to the smoothing effect on arrivals from using average arrival rates in the simulation. However, even across different arrival sets, implementing a fast track had a significant and consistent effect on median wait times, as shown in Table 3, which is ordered from the greatest to least percent reduction in median wait time.

Within a typical scenario, the addition of a fast track lowered median wait times for ESI level 4 and 5 patients. If an additional nurse was added to cover the fast track, the median wait time was also lower for ESI-3 patients. If the fast track nurse was reassigned from the main ED, the median wait time for ESI-3 patients increased slightly (see Table 4).

**Figure 4:**
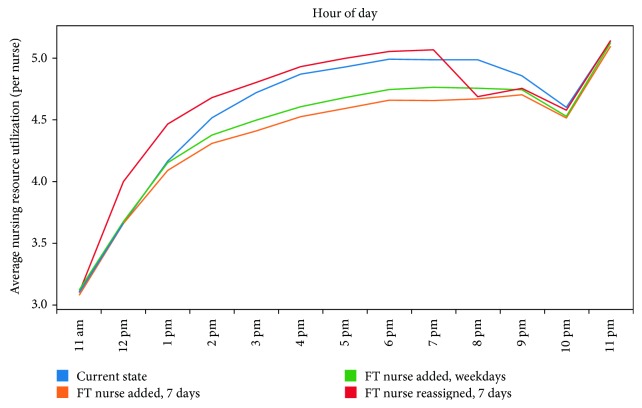
Average nursing resource demand by hour during hours of fast track operation.

Figure 4 describes the average nursing resource demand or the average total number of simulation providers required at each hour of the day divided by the number of nurses on staff at that time. The fast track reduced the nursing demand during hours a nurse is added in the fast track. Reassigning one of the ED nurses to the fast track resulted in a very slight increase in nursing resource demand.

## 4. Discussion

This report describes a novel modeling approach to provide data to support fast track implementation decision making in an ED. In the model's simulation of our institution, the results indicated that the addition of a fast track during peak ED patient volume hours may reduce median patient wait times by approximately 35 percent and over 70 percent for the ESI-4 and ESI-5 patients. Lower wait times correlated with improved ED throughput overall. However, the increased throughput did not correlate with significant increased nursing resource demand. Interestingly, the simulated maximum wait times were not significantly different for the fast track implementation scenario compared to the current state. This observation may indicate that while fast track implementation benefits throughput in usual operations, it may not be effective on its own as a solution for extreme crowding in the ED.

Based on the results of this novel modeling approach, we have begun work to implement a fast track at the campus A hospital. We plan to open the unit during the observed times of high census. It is anticipated to contain five beds, requiring the addition of one nurse. An advanced practice provider will shift to the area during times that it is open. Some ESI-3 patients may also be considered for the area, dependent upon patient characteristics and the current occupancy of the area. Medications and other supplies will be moved within the unit to be easily accessible.

Based on our experience, we believe our modeling strategy to be a promising mechanism to support fast track implementation decision making. While our model has only been studied at a single site, the model's flexibility enables it to be generalized to other EDs. In this regard, the model does have some limitations that warrant discussion. The model necessarily includes some assumptions; however, these generally were designed to conservatively impact results. First, the model assumes that the distribution of service times, as fit to historical data, remains the same. In practice, it is likely that separating low- and high-acuity patients will lead to greater efficiency in the treatment process, reducing the average service time. The model also does not account for the flexibility the ED staff has in choosing to which unit patients should go and when a patient should be admitted. In the model, this resulted in a few outlier cases of fast track patients with very high wait times. In practice, ED staff would have the flexibility to balance the load between the two units. In other words, ED staff may direct low-acuity patients to the main ED if it is less busy than the fast track on a given day, or they may direct some ESI-3 cases to the fast track for care, if necessary.

It is important to note that a potential limitation of our model's generalizability is that it did not directly include physician and advanced practice provider resources for practical reasons described previously. Based on the ED leadership's assessment of the current state at the time of this study, no additional independent providers were anticipated to be required should a fast track be opened, only that one may be shifted to that area within the current provider staffing model. Therefore in this specific hospital study, the limiting personnel factor in the model was the number of nurses. At other institutions in which the staffing constraints differ from the case site (or if the staffing constraints change at this institution in the future), the model may be adjusted easily to reflect whichever group or, if necessary, groups of staff act as a limiting factor. The limiting resource method we have proposed allows one to study the queue dynamics without allowing the results to be overly influenced by the specific personnel constraints of any one hospital. For hospitals that may require multiple personnel constraints to be considered, the queueing model may be applied in successive phases, with a number of providers equivalent to each limited resource, to preserve its ease of build and analysis. We believe that the principles of our novel modeling technique are generalizable, given the feasibility of simulation parameter adjustments reflecting local personnel resource constraints.

Another potential limitation of our modeling was that it did not directly account for the potential for variability in the number of patients per hour seen in the main ED versus the fast track area. While it is intuitive that patients with lower acuity will likely have faster turnover, we had no prior experience upon which to estimate a differential patient per hour turnover. In the absence of data to estimate this, we felt that the variable number of providers required to care for a patient based on ESI score as reported by ED staff was a suitable surrogate for this effect in our modeling.

While this model uses a similar structure to prior discrete event simulations and notwithstanding the limitations discussed, it offers additional insight into the effects of a fast track installation, particularly with regard to nursing resource demand. Previous studies saw a reduction in wait times of approximately 20–25 percent [18, 23], while ours saw a greater reduction which may be due to differences in patient population. Additionally, our model had the additional ability to evaluate nursing resource demand based on patient acuity, which those models did not analyze beyond bed capacity. When compared to previous agent-based simulations of the emergency department, its basis in queueing theory enabled this model to have a more simple structure that does not necessarily require timing each individual step of the patient care process for each agent but instead allows it to be grouped into a few large queues. This simplified queueing structure allows the model to be easily disseminated and replicated in other hospital settings.

## 5. Conclusions

With the influx of patients from growing populations and expanding health care coverage into EDs, hospitals face pressure to efficiently use and adapt existing resources while maintaining quality of care. Mathematical modeling and simulation offer efficient, relatively inexpensive methods to inform and evaluate proposed solutions to crowding, such as a fast track for low-acuity patients. While a number of studies have measured the impact of a fast track at a case site through implementation, these cases may not transfer easily to other hospitals seeking to make a similar decision. In previous simulation studies, the models have effectively analyzed the effect of the fast track on wait times, but did not explore how increased throughput affected ED staff resource demand.

In this paper, we have expanded the simple queueing model with discrete event simulation, accounting for stochastic arrival rates and service times, which enabled it to calculate wait times. More importantly, the model evaluated the space available in the ED based on the current nursing resource demand, determined using the ESI levels of patients. The results indicated that the implementation of a fast track can reduce patient wait times without increasing nursing resource demand.

Future work may be done in exploring and modeling “worst case” scenarios and potential solutions. While the queueing model presented here is relatively simple and easy to apply to other hospitals, the model may be expanded by adding additional units or dividing up the service time into smaller blocks such as bedside registration, treatment, and transportation to radiology. As the model's foundation is in queueing theory, it may easily be applied to other health care settings that involve lines to see service providers, particularly in cases where one provider services multiple patients at one time.

This study provides evidence that a queue-based simulation serves as a useful tool in evaluating the need and potential impact of implementing a fast track in a hospital ED on patient wait times and quality of service. This modeling approach may easily be applied to other hospitals nationwide in order to evaluate changes to the ED without the cost and time of a physical case study.

## Figures and Tables

**Table 1:** Provider-to-patient ratios.

Patient ESI level	Number of providers
ESI-1	5
ESI-2	4
ESI-3	2
ESI-4	1
ESI-5	1

**Table 2:** Population metrics.

	Non-LWBS count (%)	LWBS count (%)	Total count (%)
ESI-1	78 (0.20%)	0 (0%)	78 (0.20%)
ESI-2	6958 (17.77%)	32 (0.08%)	6990 (17.86%)
ESI-3	19477 (49.75%)	317 (0.81%)	19794 (50.56%)
ESI-4	9954 (25.43%)	243 (0.62%)	10197 (26.05%)
ESI-5	2015 (5.15%)	72 (0.18%)	2087 (5.33%)
Total	38482 (98.30%)	664 (1.70%)	39146 (100%)

**Table 3:** Average percent reduction in observed median wait time from current state.

Scenario	Average percent reduction (%) in median wait time ± SD
FT nurse added, 7 days, 12 pm–8 pm	35.8 ± 2.2
FT nurse added, weekdays, 12 pm–8 pm	29.1 ± 2.2
FT nurse reassigned, 7 days, 12 pm–8 pm	24.6 ± 2.3
Additional ED nurse in ED, 7 days, 12 pm–8 pm	13.6 ± 2.4
FT nurse added, Mondays, 12 pm–8 pm	8.9 ± 2.3

**Table 4:** Median wait time by ESI and scenario.

Scenario	ESI-3	ESI-4	ESI-5
Current state	21.46	16.46	16.73
FT nurse added, 7 days, 12 pm–8 pm	14.30	4.12	4.41
FT nurse added, weekdays, 12 pm–8 pm	16.55	5.27	5.49
FT nurse reassigned, 7 days, 12 pm–8 pm	21.73	4.25	4.73

## Appendix

### A. Building the Model in MATLAB

For the initial registration queue, a vector of time between arrivals was generated using a time-dependent Poisson process. This vector was then transformed into a vector of all arrival times relative to *t* = 0. ESI was probabilistically assigned to each arrival in a corresponding vector. Based on the ESI of each arrival, a vector of corresponding physician service times was generated. All arrivals then used a traditional first-in, first-out queueing model [17] to calculate arrival times to their assigned ED queue, as determined by ESI, time of day, and day of week.

The fast track queue operated as a simple first-come, first-served (FCFS) queue with one nurse, or the equivalent of five service providers. Using the given vectors of arrivals and service times, the model could progress through each arrival and calculate the patient's wait time. Wait time was calculated by sorting a vector of departure times associated with each service provider, then updating the departure times for the next available provider(s), with the number of providers required as determined by ESI.

The regular ED queue was FCFS by priority as determined by ESI. It operated much like the fast track queue, except the program also tracks the set of patients waiting, and sorts this vector by ESI before their departure times are calculated.

### B. Mathematical Methodology of Poisson Process Arrivals

The queueing systems assumed the patient population to be infinite. Arrivals to the registration queue followed a nonstationary Poisson process with arrival rate *λ*(*t*), and service times were exponentially distributed with average *μ* = 5 minutes. In the traditional queueing model, *λ* is not time dependent, but this was adjusted to account for the hospital's hourly arrival curve. ESI score was assigned by historical probability. The arrivals to the ED/FT queues followed after the departure from registration with a short delay, and the service times followed a gamma distribution with shape *k*(*e*) and scale *θ*(*e*), which are dependent on the patient's ESI score.

Let *T* be the random time between successive arrivals at the triage/registration queue [19]. The cumulative distribution function of *T* is given by

(B.1) $$\begin{array}{c} \mathbb{P}\left\{T\leq t\right\}=1-exp\left(-\lambda t\right). \end{array}$$

Let exponential random variables *X*_1_, *X*_2_,… represent the time between arrivals. The *i*th arrival is *A*_*i*_ = *A*_*i*−1_ + *X*_*i*_. When *X*_*i*_ is simulated using uniform random variable *U*_*i*_,

(B.2) $$\begin{array}{c} X_{i}=-\frac{ln\left(U\right)}{\lambda }. \end{array}$$

Therefore, each arrival time *A*_*i*_ was described as

(B.3) $$\begin{array}{c} A_{i}=A_{i-1}+X_{i} \end{array}$$

for *i* > 0 and where *A*_0_ = 0.
